# Why efficient bifunctional hydrogen electrocatalysis requires a change in the reaction mechanism

**DOI:** 10.1016/j.isci.2024.108848

**Published:** 2024-01-08

**Authors:** Samad Razzaq, Kai S. Exner

**Affiliations:** 1 University Duisburg-Essen, Faculty of Chemistry, Theoretical Inorganic Chemistry, Universitätsstraße 5, 45141 Essen, Germany; 2 Cluster of Excellence RESOLV, Bochum, Germany; 3 Center for Nanointegration (CENIDE) Duisburg-Essen, Duisburg, Germany

**Keywords:** Chemistry, Catalysis, Electrochemistry

## Abstract

Hydrogen evolution reaction (HER) and hydrogen oxidation reaction (HOR) are both two-electron processes that culminate in the formation or consumption of gaseous hydrogen in an electrolyzer or a fuel cell, respectively. Unitized regenerative proton exchange membrane fuel cells merge these two functionalities into one device, allowing to switch between the two modes of operation. This prompts the quest for efficient bifunctional electrode materials catalyzing the HER and HOR with reasonable reaction rates at low overpotentials. In the present study using a data-driven framework, we identify a general criterion for efficient bifunctional performance in the hydrogen electrocatalysis, which refers to a change in the reaction mechanism when switching from cathodic to anodic working conditions. The obtained insight can be used in future studies based on density functional theory to pave the design of efficient HER and HOR catalysts by a dedicated consideration of the kinetics in the analysis of reaction mechanisms.

## Highlights

• Microkinetic modeling of hydrogen evolution and oxidation by a data-driven approach
• Adsorption-free energy of adsorbed hydrogen is not a suitable descriptor
• General criterion for efficient bifunctional performance is identified
• A change in the reaction mechanism is accompanied with a facile Tafel kinetics

## Graphical abstract

**Figure:**
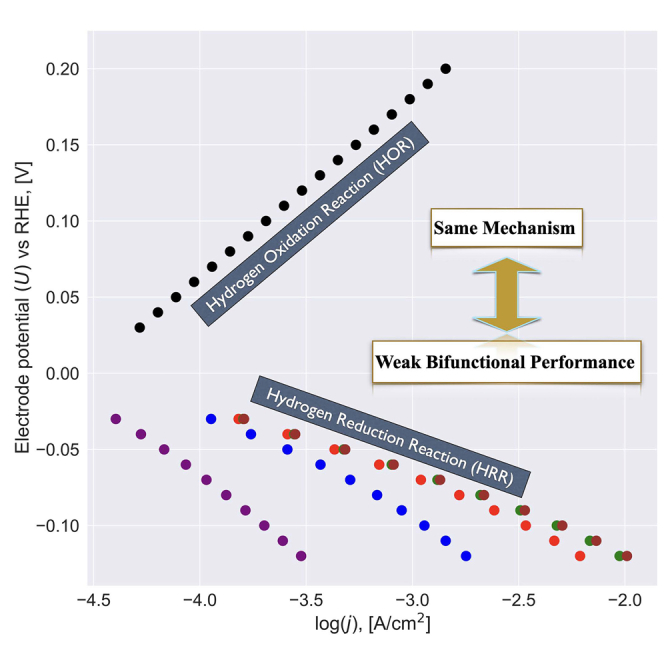

## Introduction

At the present consumption rate, global energy reserves may be depleted in less than two centuries.^1^ Therefore, a global effort has been made to explore renewable and clean alternatives to fossil fuels. Mother Nature has endowed us with abundant renewable energy sources, like solar, wind, tide, and biomass. However, the intermittent availability of these resources poses a challenge.^2^ Therefore, studies on renewable energy sources must be followed by efforts to develop dependable energy transformation and storage systems.^3^^,^^4^^,^^5^ Hydrogen production via proton exchange membrane electrolyzer (PEME)^6^^,^^7^^,^^8^ and hydrogen-to-electricity conversion through proton exchange membrane fuel cells (PEMFC)^9^^,^^10^^,^^11^ have received significant attention during the last two decades. While hydrogen evolution reaction (HER) takes place at the cathode of a PEME, hydrogen oxidation reaction (HOR) is encountered in a PEMFC. Both reactions are facile two-electron processes, 2 H^+^ + 2 e^–^ ⇌ H_2_, *U*^0^ = 0 V vs. reversible hydrogen electrode (RHE), and are not the main limitation in both devices, recalling that the four-electron oxygen evolution and oxygen reduction reactions reveal sluggish reaction kinetics.^12^^,^^13^^,^^14^^,^^15^ The present manuscript does not focus on the bottleneck of PEME and PEMFC relating to the oxygen electrocatalysis but rather addresses the hydrogen electrocatalysis by referring to the idea of combining the PEME and PEMFC components into a single system, also denoted as unitized regenerative proton exchange membrane fuel cell (UR-PEMFC).^16^^,^^17^^,^^18^ The technology’s compatibility with various renewable energy sources, as mentioned in refs.^16^^,^^17^, positions it as a cornerstone in the emerging hydrogen economy, catalyzing the transition away from fossil fuels. This is also in alignment with the broader vision of a hydrogen-based energy landscape, as envisioned by Crabtree et al*.*^4^ Moreover, the compact and modular nature of URFCs, coupled with their high energy conversion efficiency, makes them well-suited for decentralized energy production. When switching between the electrolyzer and fuel cell modes, either the HER or the HOR takes place on the same electrode in the UR-PEMFC. Consequently, efficient bifunctional materials for the hydrogen electrocatalysis are called for, and the conventional approach of identifying active electrocatalysts refers to theoretical considerations in the realm of heuristic materials screening.^19^^,^^20^^,^^21^^,^^22^^,^^23^^,^^24^^,^^25^ Since the pioneering works of Nørskov et al. at the beginning of the 21st century,^19^^,^^20^ it is a common paradigm to calculate adsorption (free) energies of intermediate species for an electrocatalytic process. Relating to the simple hydrogen electrocatalysis, the only relevant intermediate species is adsorbed hydrogen, ∗H, albeit recent works have pointed out the importance of hydroxyl groups for the HER in alkaline media.^26^

Using the computational hydrogen electrode (CHE) approach, the adsorption free energy of adsorbed hydrogen, Δ*G*_∗H_, can be readily calculated, and its value should be ideally around zero to obtain a catalytic material with high activity toward HER and HOR.^20^^,^^27^^,^^28^ While the notion of thermoneutral bonding as the thermodynamic ideal has been challenged by several scientists in recent years,^29^^,^^30^^,^^31^^,^^32^^,^^33^^,^^34^ the Δ*G*_∗H_ analysis is yet the most commonly applied method in theoretical electrochemistry to evaluate the electrocatalytic activity based on heuristics tools.^35^^,^^36^^,^^37^ One caveat of this approach, when applying it to the bifunctional hydrogen electrocatalysis, refers to the fact that the Δ*G*_∗H_ analysis tacitly assumes that the same reaction mechanism (Volmer-Heyrovsky) is operative for the HER and HOR both whereas the Δ*G*_∗H_ analysis largely ignores another mechanistic description (Volmer-Tafel) consisting of a chemical reaction step (*vide infra*). We ponder on this finding in the present manuscript by a dedicated microkinetic evaluation of the Volmer-Heyrovsky mechanism under cathodic and anodic potential conditions. Applying a data-driven framework as introduced in recent works of the authors,^38^^,^^39^^,^^40^ we are able to generalize the requirements of highly active catalysts for the bifunctional hydrogen electrocatalysis. Our analysis demonstrates that efficient bifunctional hydrogen electrocatalysis is only encountered if a switch in the reaction mechanism takes place, thus questioning the validity of the Δ*G*_∗H_ descriptor for the development of bifunctional materials.

## Results and discussion

### Reaction mechanism

In the literature, the Volmer-Heyrovsky and Volmer-Tafel mechanisms are discussed as mechanistic descriptions for the HER, and thus, for the HOR, the reverse pathways, Heyrovsky-Volmer or Tafel-Volmer, are operative.^35^^,^^41^^,^^42^^,^^43^^,^^44^^,^^45^^,^^46^^,^^47^ While the Volmer and Heyrovsky steps are of electrochemical nature due to the occurrence of proton-coupled electron transfer steps, the chemical Tafel step does not contain any charge transfer. Equations 1, 2, and 3 summarize the elementary processes; please note that ∗ indicates the active site of an electrocatalyst, and *k*_j_ refers to the rate constant for each step:

(Equation 1) $$\text{Volmer}\text{step}:*+H^{+}+e^{-}\overset{k_{1}}{\underset{k_{-1}}{⇌}}\;*H$$

(Equation 2) $$\text{Heyrovsky}\text{step}:*H+H^{+}+e^{-}\overset{k_{2}}{\underset{k_{-2}}{⇌}}*+H_{2}$$

(Equation 3) $$\text{Tafel}\text{step}:*H+*H+H^{+}+e^{-}\overset{k_{3}}{\underset{k_{-3}}{⇌}}*+H_{2}$$

The HER and HOR mechanisms for a given electrode material under acidic or alkaline conditions are still a matter of debate in the literature. For example, even for a seemingly simple single-crystalline Pt(111) surface, it is controversially discussed whether the mechanistic description of the HER refers to the Volmer-Heyrovsky or Volmer-Tafel pathway.^48^^,^^49^^,^^50^^,^^51^^,^^52^^,^^53^^,^^54^^,^^55^^,^^56^ Similarly, Zheng et al.^44^ and Sheng et al.^45^ have demonstrated that the HOR for platinum group metals occurs via the Tafel-Volmer pathway. In contrast, investigations by Strmcnik et al.^57^ predict that the Tafel-Heyrovsky pathway is encountered during the HOR.

In the present manuscript, we do not aim to resolve the mechanistic controversies of the HER and HOR over platinum group metals but rather discuss mechanistic implications relating to the bifunctional hydrogen electrocatalysis on the atomic scale. To do so, we make use of the concept of free-energy diagrams (FED),^56^^,^^58^ which condense the thermodynamic and kinetic information of an electrocatalytic process in a single framework.

### Free-energy diagram

**Figure 1:**
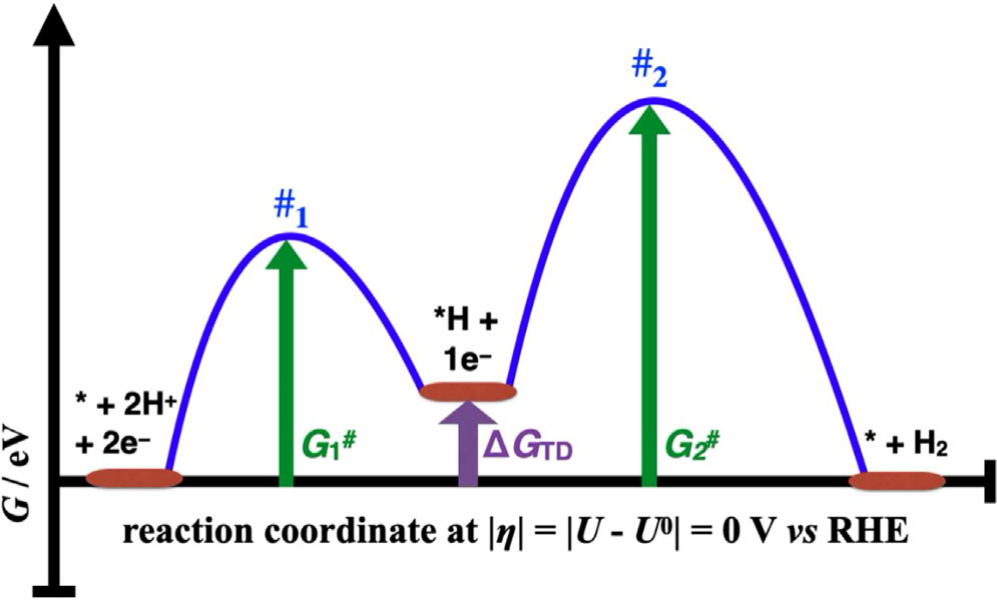
Free-energy diagram of a two-electron process under equilibrium conditions Free-energy diagram (FED) for a two-electron process at an overpotential of *η = U – U*^0^ = 0 V. Δ*G*_TD_ denotes the free-energy change for the formation of the reaction intermediate, ∗H (thermodynamics), whereas *G*_1_^#^ and *G*_2_^#^ are the transition-state free energies of the Volmer (#_1_) and Heyrovsky (#_2_) steps (kinetics), respectively.

Figure 1 illustrates a prototypical example of a generalized FED of the HER following the Volmer-Heyrovsky mechanism. Accordingly, the free-energy landscape of the HOR for the same active site is given by reading the FED from right to left. The thermodynamic information of the FED is given by the free energies of the active site, ∗ +2 H^+^ + 2e^–^, the reaction intermediate, ∗H + 1 H^+^ + 1e^–^, and the product state, ∗ + H_2_, culminating into the definition of the binding strength of the ∗H precursor, denoted as Δ*G*_∗H_ = Δ*G*_TD_. The kinetic information refers to the transition-state free energies *G*_1_^#^ and *G*_2_^#^, indicating the free energy of activation for the Volmer and Heyrovsky steps with respect to the active site, respectively. We would like to emphasize that we have arbitrarily chosen Δ*G*_TD_ > 0 eV in this figure, albeit it is possible that Δ*G*_TD_ < 0 eV is met. In this case, the reaction intermediate, ∗H, is used as the initial configuration in the FED**,** followed by a renumbering of the electron-transfer steps in the mechanistic description.^23^^,^^56^^,^^58^^,^^59^ Therefore, it is sufficient to inspect positive values of Δ*G*_TD_ since the renumbering procedure results in an FED with a positive free-energy change relating to the first elementary step.^23^ Similarly, we have arbitrarily chosen *G*_1_^#^ < *G*_2_^#^ in the FED of Figure 1, whereas the opposite, *G*_1_^#^ > *G*_2_^#^, may also hold true. To explore the phase space of various opportunities relating to the transition-state free energies *G*_1_^#^ and *G*_2_^#^, we use a data-driven strategy,^38^^,^^39^^,^^40^ as explained in a later subsection, to take this fact into account.

In experimental investigations of the hydrogen electrocatalysis,^52^^,^^60^^,^^61^ the logarithm of the current density, *j*, is evaluated as a function of the applied electrode potential, *U*, also denoted as Tafel plot.^62^ It is noteworthy that the FED of Figure 1 is potential dependent, and we can translate the energetics of the elementary steps to anodic or cathodic potential conditions when considering the number of electrons transferred for each process.^56^^,^^58^ To bridge the gap between the microscopic picture of theoretical electrochemistry (cf. Figure 1) and the macroscopic world of experimental investigations, we apply a microkinetic approach as introduced recently^63^^,^^64^ so that Tafel plots for the bifunctional hydrogen electrocatalysis based on the Volmer-Heyrovsky mechanism are derived. The following subsection shortly summarizes the microkinetic framework to depict the FED in the form of log *j* = f(*U*).

Any electrochemical process requires an overpotential (|*η|*) to proceed spontaneously. For the HER, commonly, applied overpotentials on the order of 50–100 mV are required to reach a reasonable current density of 10 mA/cm^2^,^65^^,^^66^ corresponding to a solar-to-hydrogen conversion efficiency of about 10%.^67^ For the modeling of electrochemical processes, density functional theory (DFT) computations require a formalism that considers a constant electrode potential (referred to as grand-canonical description).^68^ However, it is still common to use DFT in the traditional manner, which assumes a constant charge (known as canonical description) rather than a constant potential.^27^ While grand-canonical methodologies are of high relevance for the development of the scientific discipline of theoretical electrochemistry, their detailed discussion is outside the scope of our current study. When constructing or analyzing the potential dependence of free-energy diagrams, we reply on a canonical approach in that the free energies of intermediate and transition states reveal a constant shift with increasing overpotential. This appears to be justified since the dipole moment of the reaction intermediate, ∗H, is moderate, and thus, the canonical description can be used for the evaluation of the energetics.^69^

### Microkinetic approach

Microkinetic investigations are commonly built on either the quasi-equilibrium or the steady-state assumptions.^70^ While the quasi-equilibrium approach relies on the approximation of a nearly constant coverage, *θ*, of the reaction intermediate, ∗H, at any arbitrary electrode potential, the steady-state assumption is somewhat more elaborated because it allows for varying coverages in dependence of applied bias.^71^^,^^72^^,^^73^^,^^74^^,^^75^ To derive the relationship between the applied electrode potential and the current density under steady-state conditions, first, we define the reaction rate for the HER according to Equation 4^63^:

(Equation 4) $$r\left(\eta \right)=\frac{d\left[H_{2}\right]}{dt}=k_{2}\left(\eta \right)\cdot \theta_{*H}\left(\eta \right)-k_{-2}\left(\eta \right)\cdot \theta_{*}\left(\eta \right)$$

In Equation 4, $\theta_{*}$, $\theta_{*H}$, $k_{2}$, and $k_{-2}$ denote the coverage of the active site, coverage of the intermediate species, the rate constant of the second elementary step, and rate constant of the reverse second elementary step, respectively. In the case of the Volmer-Heyrovsky mechanism for the FED of Figure 1, $k_{2}$ is encountered with the Heyrovsky step, whereas $k_{-2}$ refers to the reverse Heyrovsky step (cf. Equation 2). The applied overpotential, *η*, is the difference between the actual electrode potential, *U*, and the equilibrium potential of the HER and HOR; that is, *U*^0^ = 0 V vs. RHE. Please note that the absolute value of *η* is used throughout the analysis (cf. Figure 1).

The coverage of the intermediate adsorbed hydrogen, $\theta_{*H}$, can be obtained as a solution of the following differential equation (cf. Equation 5):

(Equation 5) $${\left(\frac{d\theta_{*H}}{\text{dt}}\right)}_{\eta }={+k}_{1}\left(\eta \right)\cdot \theta_{*}\left(\eta \right){\;-\;k}_{-1}\left(\eta \right)\cdot \theta_{*H}\left(\eta \right)\;{-k}_{2}\left(\eta \right)\cdot \theta_{*H}\left(\eta \right)\;{+k}_{-2}\left(\eta \right)\cdot \theta_{*}\left(\eta \right)$$

In Equation 5, $k_{1}$, and $k_{-1}$ refer to the rate constants of the Volmer and reverse Volmer steps (cf. Equation 1), respectively. Considering the balance of active sites (cf. Equation 6) and steady-state conditions (cf. Equation 7), Equation 5 translates to Equation 8^63^:

(Equation 6) $$\theta_{*}\left(\eta \right)+\theta_{*H}\left(\eta \right)=1$$

(Equation 7) $${\left(\frac{{d\theta }_{*H}}{dt}\right)}_{\eta }=0$$

(Equation 8) $$\theta_{*H}\left(\eta \right)=\frac{k_{1}\left(\eta \right)+k_{-2}\left(\eta \right)}{k_{1}\left(\eta \right)+k_{-1}\left(\eta \right)+k_{2}\left(\eta \right)+k_{-2}\left(\eta \right)}$$

Correspondingly, the coverage of the active site is given by Equation 9:

(Equation 9) $$\theta_{*}\left(\eta \right)=\frac{k_{-1}\left(\eta \right)+k_{2}\left(\eta \right)}{k_{1}\left(\eta \right)+k_{-1}\left(\eta \right)+k_{2}\left(\eta \right)+k_{-2}\left(\eta \right)}$$

Taking Equations 4, 8, and 9 into account, the reaction rate can be expressed as follows:

(Equation 10) $$r\left(\eta \right)=\frac{k_{1}\left(\eta \right)\cdot k_{2}\left(\eta \right)}{k_{1}\left(\eta \right)+k_{-1}\left(\eta \right)+k_{2}\left(\eta \right)+k_{-2}\left(\eta \right)}-\;\frac{k_{-1}\left(\eta \right)\cdot k_{-2}\left(\eta \right)}{k_{1}\left(\eta \right)+k_{-1}\left(\eta \right)+k_{2}\left(\eta \right)+k_{-2}\left(\eta \right)}$$

The rate constants of the elementary steps are defined according to transition-state theory by Equations 11, 12, 13, and 14,^63^:

(Equation 11) $$k_{1}\left(\eta \right)=\frac{k_{B}\cdot T}{h}\cdot e^{\frac{-{\Delta G}_{1}^{\#}}{k_{B}\cdot T}}\cdot e^{\frac{\alpha_{1}\cdot e\cdot n}{k_{B}\cdot T}}$$

(Equation 12) $$k_{-1}\left(\eta \right)=\frac{k_{B}\cdot T}{h}\cdot e^{\frac{-{(\Delta G}_{1}^{\#}-{\Delta G}_{\text{TD}})}{k_{B}\cdot T}}\cdot e^{\frac{-\left(1-\alpha_{1}\right)\cdot e\cdot n}{k_{B}\cdot T}}$$

(Equation 13) $$k_{2}\left(\eta \right)=\frac{k_{B}\cdot T}{h}\cdot e^{\frac{-{\Delta G}_{2}^{\#}}{k_{B}\cdot T}}\cdot e^{\frac{\alpha_{2}\cdot e\cdot n}{k_{B}\cdot T}}$$

(Equation 14) $$k_{-2}\left(\eta \right)=\frac{k_{B}\cdot T}{h}\cdot e^{\frac{-{(\Delta G}_{2}^{\#}+{\Delta G}_{\text{TD}})}{k_{B}\cdot T}}\cdot e^{\frac{-\left(1-\alpha_{2}\right)\cdot e\cdot n}{k_{B}\cdot T}}$$

In Equations 11, 12, 13, and 14, $\alpha_{1}$ and $\alpha_{2}$ are the transfer coefficients of the Volmer and Heyrovsky steps, respectively. The values ${\Delta G}_{1}^{\#}$ and ${\Delta G}_{2}^{\#}$ refer to the free-energy barriers of the Volmer and Heyrovsky steps, respectively, which are related to the transition-state free energy of the respective step by Equations 15 and 16:

(Equation 15) $$G_{1}^{\#}={\Delta G}_{1}^{\#}$$

(Equation 16) $$G_{2}^{\#}={{\Delta G}_{\text{TD}}+\Delta G}_{2}^{\#}$$

Subsequently, the reaction rate is can be translated into the current density, *j*^63^:

(Equation 17) $$j\left(\eta \right)=e\cdot z\cdot r\left(\eta \right)\cdot \Gamma_{\text{act}}$$

In Equation 17, *z* corresponds to the number of transferred electrons (*z* = 2), and $\Gamma_{\text{act}}$ indicates the number of active sites on the catalyst surface per cm^2^ of surface area.

Using Equation 17 in conjunction with Equations 11, 12, 13, and 14, the current density in dependence of the applied overpotential for the steady-state approach reads^63^:

(Equation 18) $$j\left(n\right)=\frac{2\cdot k_{B}\cdot T\cdot e\cdot \Gamma_{\text{act}}\;}{h}\cdot \frac{\exp\left(\frac{\left(\alpha_{1}+\alpha_{2}\right)\cdot \eta \cdot e}{k_{B}\cdot T}\right)-\exp\left(\frac{-\left({2-\alpha }_{1}-\alpha_{2}\right)\cdot \eta \cdot e}{k_{B}\cdot T}\right)}{\exp\left(\frac{G_{1}^{\#}+\alpha_{2}\cdot \eta \cdot e}{k_{B}\cdot T}\right)+\exp\left(\frac{G_{2}^{\#}-\;{\Delta G}_{\text{TD}}+\alpha_{1}\cdot \eta \cdot e}{k_{B}\cdot T}\right)+\exp\left(\frac{G_{1}^{\#}-\;{\Delta G}_{\text{TD}}-\left(1-\alpha_{2}\right)\cdot \eta \cdot e}{k_{B}\cdot T}\right)+\exp\left(\frac{G_{2}^{\#}-\left(1-\alpha_{1}\right)\cdot \eta \cdot e}{k_{B}\cdot T}\right)}$$

The second term in the numerator of Equation 18 corresponds to the backward reactions of the mechanistic description of a two-electron processes. Considering that in the Tafel regime, |*η|* > 0.03 V,^74^^,^^75^^,^^76^ the backward reaction can be neglected, and taking into account that one of the terms in the denominator can be skipped,^63^ we arrive at the final expression for the steady-state current density, *j*, in dependence of the applied overpotential, *η* (cf. Equation 19):

(Equation 19) $$j\left(n\right)=\frac{2\cdot k_{B}\cdot T\cdot e\cdot \Gamma_{\text{act}}\;}{h}\cdot \frac{\exp\left(\frac{\left(\alpha_{1}+\alpha_{2}\right)\cdot \eta \cdot e}{k_{B}\cdot T}\right)}{\exp\left(\frac{G_{1}^{\#}+\alpha_{2}\cdot \eta \cdot e}{k_{B}\cdot T}\right)+\exp\left(\frac{G_{2}^{\#}-\left(1-\alpha_{1}\right)\cdot \eta \cdot e}{k_{B}\cdot T}\right)+\exp\left(\frac{G_{2}^{\#}-\;{\Delta G}_{\text{TD}}+\alpha_{1}\cdot \eta \cdot e}{k_{B}\cdot T}\right)}$$

where $k_{B}$, T, e, $\Gamma_{\text{act}}$ and h denote the Boltzmann constant, temperature in Kelvin, elementary charge constant, number of active sites per cm^2^, and the Planck constant, respectively. Furthermore, $\alpha_{1}$ and $\alpha_{2}$ represent the transfer coefficients associated with the first and second step, respectively, while $G_{1}^{\#}$ and $G_{2}^{\#}$ correspond to the transition state free energies of the first and second step, respectively (cf. Figure 1). The peculiarity of Equation 19 refers to the fact that the FED of Figure 1 provides almost all parameters for this equation and the subsequent analysis. For a dedicated derivation of Equations 18 and 19, we refer to a recent work of one of the authors where all assumptions and approximations are discussed in depth.^63^ Please note that our microkinetic model relies on the tacit assumption of a transmission coefficient *κ* = 1.

### Data-driven analysis of Tafel plots

In our previous works, we have introduced a methodology of data-driven analyses of electrocatalytic processes over solid-state electrodes. While this approach has been exerted to the oxygen evolution,^38^^,^^39^ oxygen reduction,^40^ and nitrogen reduction reactions,^77^ herein, we apply it to the bifunctional hydrogen electrocatalysis. The procedure is as follows in that we define a basis set for the parameter space associated with Equation 18; that is, the transition-state free energies $G_{1}^{\#}$ and $G_{2}^{\#}$ as well as the adsorption free energy of adsorbed hydrogen, Δ*G*_TD_ (cf. FED of Figure 1). Following recent works on highly active HER catalysts,^33^^,^^47^^,^^49^^,^^56^^,^^58^^,^^78^^,^^79^ the limiting step of the HER reveals a transition-state free energy on the order of about 0.7 eV. Therefore, we define $G_{1}^{\#}$ = $G_{2}^{\#}$ = [0.70, 0.75, 0.80] and Δ*G*_TD_ = [0, 0.10, 0.20, 0.30, 0.40] eV, recalling that electrode materials with a binding energy deviating significantly from zero (Δ*G*_TD_ > 0.40 eV) are considered to be inactive. Given that the analysis is executed within the Python programming language, $G_{j}^{\#}$ and Δ*G*_TD_ can be expressed as [0.70, 0.80; 0.05] eV and [0, 0.40; 0.10] eV, correspondingly. Please note that the first two terms in the rectangular brackets indicate the start and stop values, whereas the value following “; ” denotes the step size between any two consecutive values. By the above-defined dataset, the parameter space of the HER is reasonably sampled. We want to emphasize that this parameter space also describes the Heyrovsky-Volmer pathway of the HOR in that $G_{2}^{\#}$ and $G_{1}^{\#}$ refer to transition-state free energies of the first and second steps in the HOR mechanism, respectively.

Developing a precise quantitative microkinetic model for the hydrogen evolution reaction (HER) and the hydrogen oxidation reaction (HOR) is an active area of research, thereby focusing solely on the activity (product formation) and neglecting potential degradation processes under cathodic reaction conditions. Recent works aimed to improve the theoretical description of the hydrogen electrocatalysis by determining the charge transfer coefficient and potential-dependent reaction barriers by grand-canonical schemes to forecast polarization curves.^80^ On the contrary, our preference is to adopt a fundamental basis set for all these values, aiming to elucidate overarching patterns within the field of hydrogen electrocatalysis by data-driven analyses. Furthermore, by adopting a microkinetic model there is no scaling relation^81^^,^^82^ assumed between the transition state and hydrogen adsorption-free energies in the given parameter range; that is, we do not need to make use of a BEP relation that connects the thermodynamic (Δ*G*_TD_) with the kinetic ($G_{1}^{\#}$ or $G_{2}^{\#}$) information, though the traditional thermodynamic analysis by assessing the hydrogen adsorption energy is governed by a BEP relation.

Hence, we define basis sets for the transfer coefficients of the Volmer and Heyrovsky steps, $\alpha_{1}$ and $\alpha_{2}$, respectively, the applied overpotential, *η*, and the density of active surface sites, $\Gamma_{\text{act}}$. We choose $\alpha_{1}$ = $\alpha_{2}$ = [0.50] without any variation since a recent microkinetic modeling work on the HER has indicated that a reasonable variation of the transfer coefficients does not impact the obtained Tafel slope qualitatively.^83^ Similarly, we choose $\Gamma_{\text{act}}$ = [5 × 10^14^] cm^−2^ since an alteration of $\Gamma_{\text{act}}$ does not change the Tafel slope; rather, it has implications on the obtained current density. The (absolute value of the) applied overpotential refers to the range of *η* = [0.03, 0.20; 0.01] V, recalling that for highly active HER catalysts, overpotentials of about 50–100 mV are already sufficient to reach a current density on the order of 10 mA/cm^2^.

We have written a Python code where we have incorporated the aforementioned details, namely for the input grid (*η*; $G_{1}^{\#}$; $G_{2}^{\#}$; ${\Delta G}_{\text{TD}}$; $\alpha_{1}$; $\alpha_{2}$; $\Gamma_{\text{act}}$), the code uses Equation 19 to calculate the current densities for the HER and HOR, which are saved in a data frame ($j_{\text{her}}$; $j_{\text{hor}}$) with a column for each variable. In section 1 of the supplemental, we have provided a pseudo script of our procedure.

**Figure 2:**
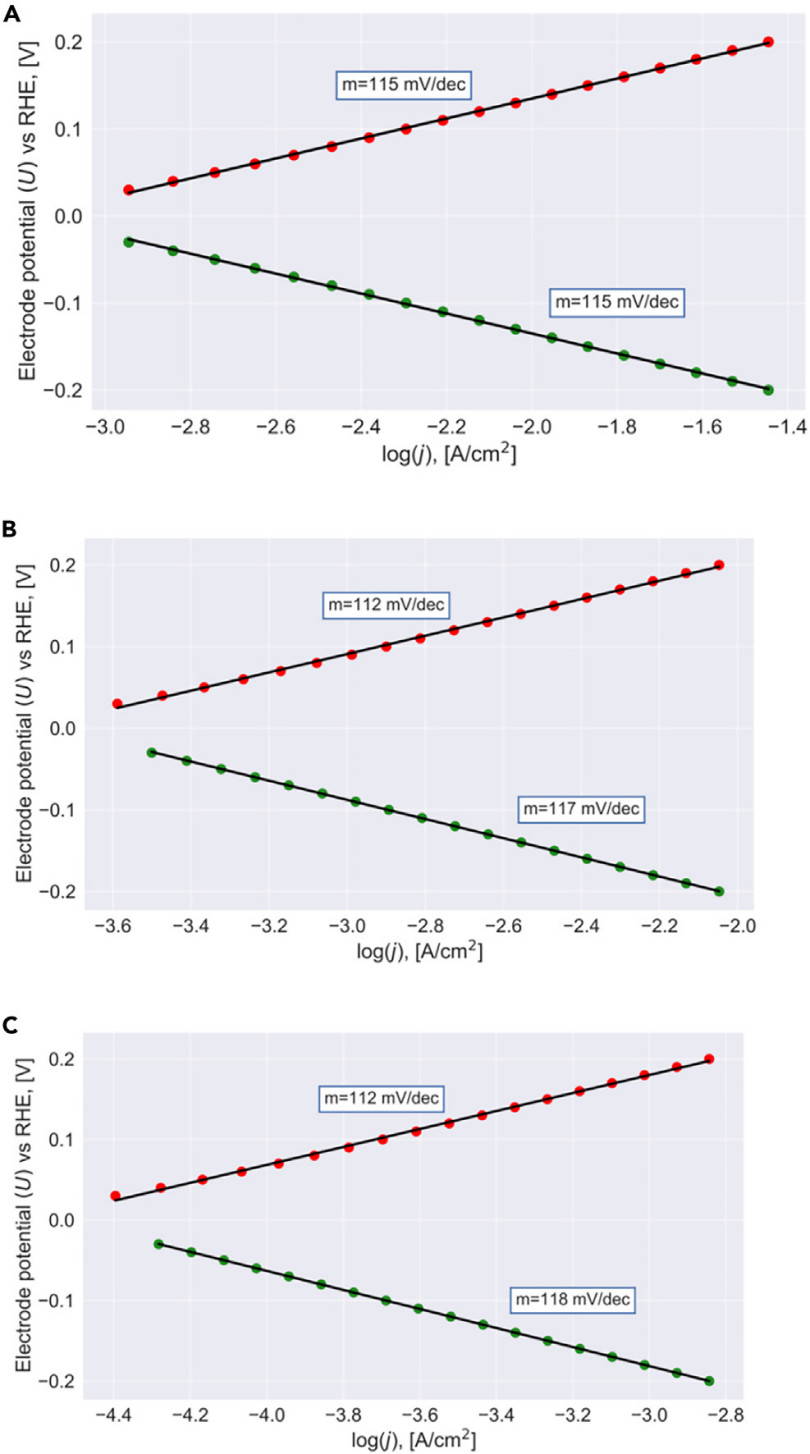
Tafel plots for a thermoneutral catalyst Steady-state analysis (cf. Equation 19) is used to convert the free-energy landscape of Figure 1 for a dataset of various free energies into a Tafel plot. Here, we depict the results for ${\Delta G}_{\text{TD}}$ = 0 eV and (A) $G_{1}^{\#}$ = 0.70 eV, $G_{2}^{\#}$ = 0.70 eV, (B) $G_{1}^{\#}$ = 0.75 eV, $G_{2}^{\#}$ = 0.70 eV, (C) $G_{1}^{\#}$ = 0.80 eV, $G_{2}^{\#}$ = 0.70 eV. HER and HOR are indicated by green and red data points, respectively. Independent of the actual energetics, a single Tafel slope is observed in the entire potential regime.

The current densities of the HER and HOR from the data frame are translated to their logarithmic values, log $j_{\text{her}}$ and log $j_{\text{hor}}$, respectively, and the applied overpotential is plotted as a function of these logarithmic values to meet the Tafel equation, *η* ∼ log *j*. Figure 2 illustrates the results of this procedure for thermoneutral bonding of adsorbed hydrogen, Δ*G*_TD_ = 0 eV, and different transition-state free energies $G_{1}^{\#}$ and $G_{2}^{\#}$. In all three cases, linear Tafel plots with a slope of about 110–120 mV/dec. are observed for the HER and HOR both. This finding is surprising since Δ*G*_TD_ = 0 eV is considered traditionally as the optimum binding energy, yet the microkinetic analysis reveals that the kinetics of materials revealing thermoneutral bonding is hampered due to a large Tafel slope. Our results are in agreement with recent experimental investigations combined with a machine-learning algorithm of Ooka et al. on the HER over platinum, reporting that Pt binds adsorbed hydrogen weakly rather than thermoneutral.^32^

**Figure 3:**
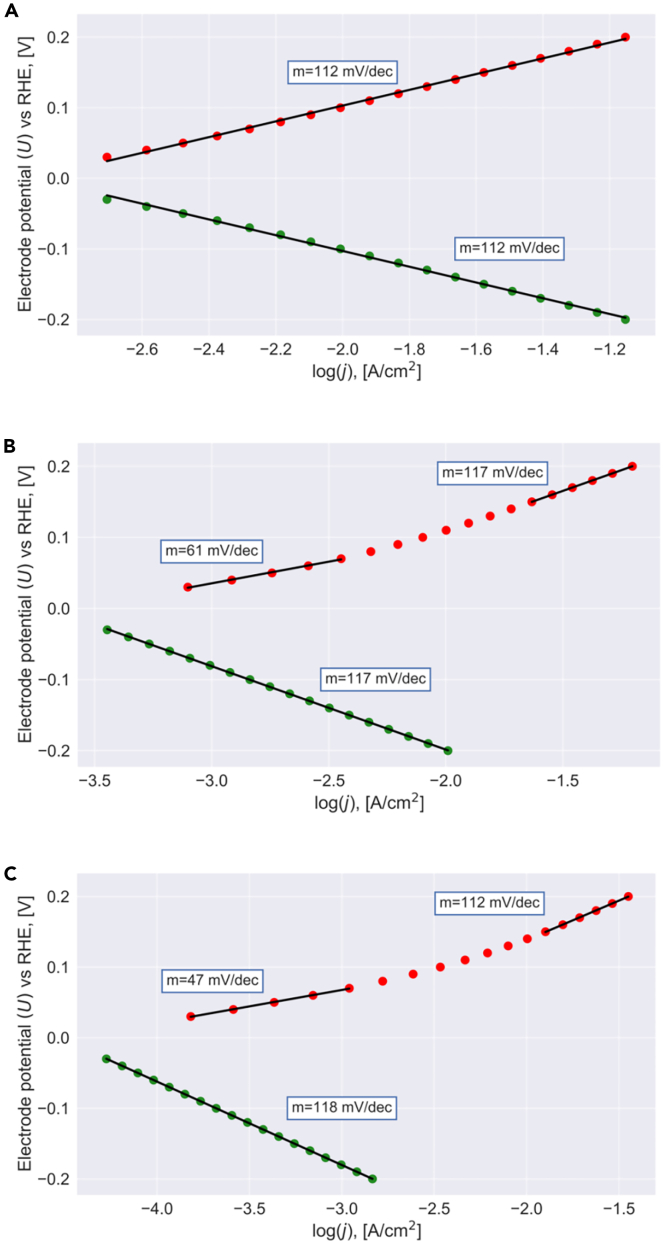
Tafel plots for a non-thermoneutral catalyst Steady-state analysis (cf. Equation 19) is used to convert the free-energy landscape of Figure 1 for a dataset of various free energies into a Tafel plot. Here, we depict the results for ${\Delta G}_{\text{TD}}$ = 0.10 eV and (A) $G_{1}^{\#}$ = 0.70 eV, $G_{2}^{\#}$ = 0.70 eV, (B) $G_{1}^{\#}$ = 0.75 eV, $G_{2}^{\#}$ = 0.70 eV, (C) $G_{1}^{\#}$ = 0.80 eV, $G_{2}^{\#}$ = 0.70 eV. HER and HOR are indicated by green and red data points, respectively. Independent of the actual energetics, a single Tafel slope is encountered for the HER whereas for the HOR, a change in the Tafel slope upon increasing overpotential is observed for $G_{1}^{\#}$ > $G_{2}^{\#}$.

In the next step, we discuss the Tafel slopes of the HER and HOR for Δ*G*_TD_ > 0 eV. Figure 3 depicts the same analysis as encountered with Figure 2 for the same dataset of $G_{1}^{\#}$ and $G_{2}^{\#}$ values. While for $G_{1}^{\#}$ = $G_{2}^{\#}$ = 0.7 eV, a single Tafel slope is observed for the HER and HOR both (cf. Figure 3A), for $G_{1}^{\#}$ > $G_{2}^{\#}$, the HOR kinetics reveals a switch in the Tafel slope from about 47 mV/dec. to about 112 mV/dec. upon increasing overpotential (cf. Figures 3B and 3C), whereas the HER still reveals a single Tafel slope. This behavior contrasts with the case of Δ*G*_TD_ = 0 eV, and the different HOR kinetics can be clearly related to the fact that weak bonding of the reaction intermediate has a positive effect on the electrocatalytic activity in terms of the Tafel slope.^56^^,^^84^ Further analysis of the HER and HOR Tafel slopes in dependence of Δ*G*_TD_ is provided in the supplemental, Figures S2–S4, revealing the same qualitative result as shown in Figure 3.

### Discussion: Limitations of the study

**Figure 4:**
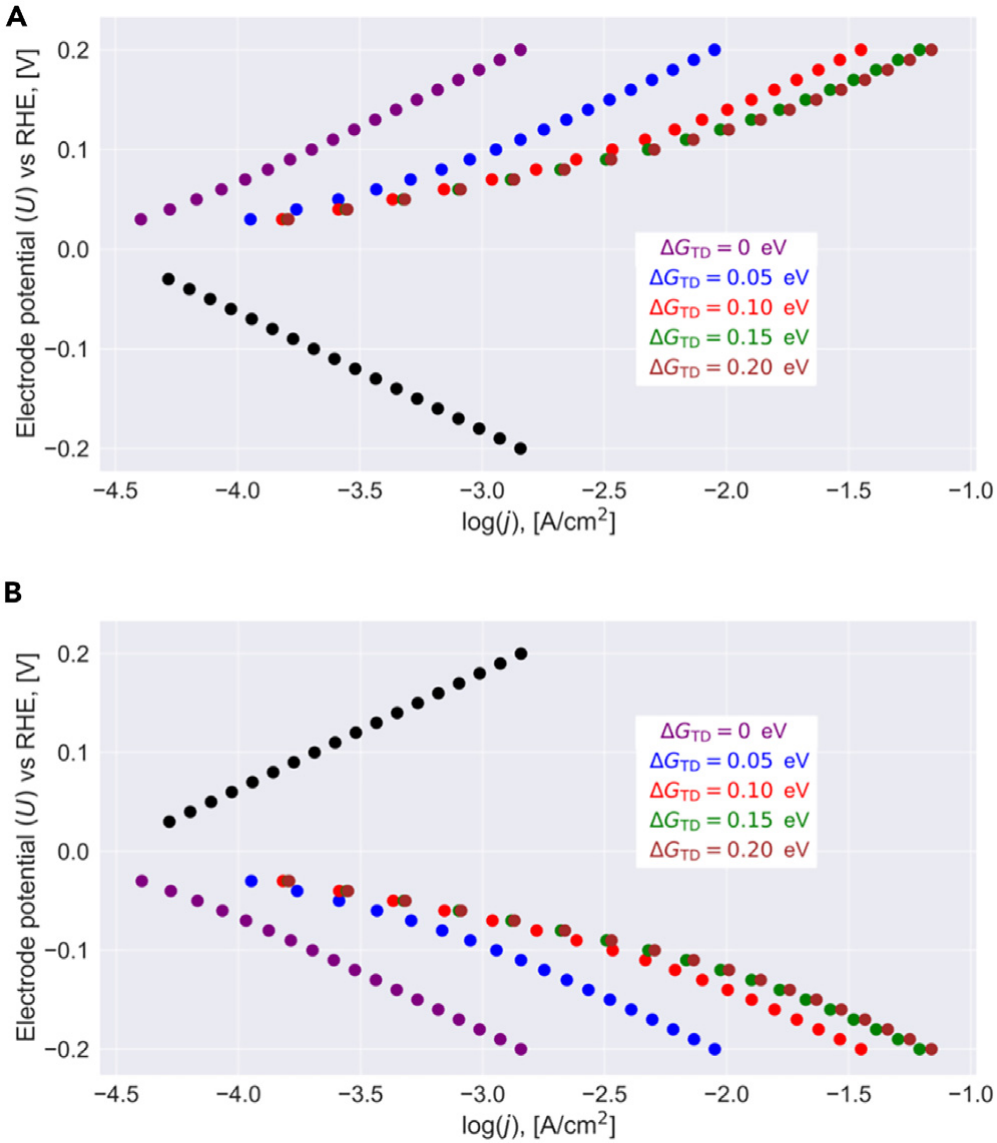
Impact of the binding energy of adsorbed hydrogen on the Tafel plot Steady-state analysis (cf. Equation 19) is used to convert the free-energy landscape of Figure 1 for a dataset of various free energies into a Tafel plot. Here, we depict the results for variable ${\Delta G}_{\text{TD}}$ and (A) $G_{1}^{\#}$ = 0.80 eV, $G_{2}^{\#}$ = 0.70 eV, (B) $G_{1}^{\#}$ = 0.70 eV, $G_{2}^{\#}$ = 0.80 eV. In case that a single Tafel slope is met independent of ${\Delta G}_{\text{TD}}$, data points are indicated in black. Otherwise, the color of the data points refers to the specified inset of ${\Delta G}_{\text{TD}}$ values.

A small Tafel slope is beneficial for the electrocatalysis since it is accompanied with a significant increase in current density upon increasing overpotential. So far, we have observed that only the HOR reveals Tafel slope smaller than 59 mV/dec. for $G_{1}^{\#}$ > $G_{2}^{\#}$ and Δ*G*_TD_ > 0 eV for overpotentials below 100 mV so that a change in the Tafel slope is encountered with increased driving force (cf. Figure 4A). In a similar fashion, we can deduce that a switch in the Tafel slope takes place for the HER if $G_{1}^{\#}$ < $G_{2}^{\#}$ and Δ*G*_TD_ > 0 eV is met (cf. Figure 4B). However, in this case, the HOR reveals a single linear Tafel regime with a slope of about 118 mV/dec. (cf. Figure 4B). Notably, weaker bonding of the reaction intermediate, adsorbed hydrogen, results in increased electrocatalytic activity (current density) for the case of an altered Tafel slope if the energetics of the transition state is kept constant. This finding illustrates the risk of using the conventional approach of adsorption-free energies in the realm of a volcano plot to predict the rates of a two-electron process.^20^^,^^27^ In summary, our data-driven analysis purports that the Tafel slope is optimized only for one of the two reaction channels, whereas for the reverse pathway, an unfavorable Tafel behavior due to a single Tafel slope is met.

In the following, we compare our results to electrochemical experiments of single-crystalline electrodes. While platinum refers to the most active electrode material in the bifunctional hydrogen electrocatalysis,^44^^,^^48^^,^^49^^,^^52^^,^^60^ an electrochemical analysis of the HER and HOR by means of polarization curves was provided by Markovich et al. already more than 20 years ago.^52^^,^^60^ In their works, the authors demonstrate that Pt(100) and Pt(110) both have two Tafel slope regimes in the HER and HOR, underpinning potential-dependent changes for both reactions. This finding cannot be explained by our microkinetic analysis based on the Volmer-Heyrovsky mechanism so that at this moment, there are two possible explanations for the deviation of our modeling approach to the experiments: (1) the reversible HER and HOR are not described by a single mechanism; or (2) the reversible HER and HOR do not occur on the same active site, but rather each electrocatalytic reaction has its own active center (e.g., top site for one reaction and hollow site for the reverse process). Following the discussion in Markovich’s work,^52^ the Pt single crystals reveal a symmetrical log *j* vs. *η* relationship with a single exchange current density for the hydrogen electrode reaction applicable to both anodic and cathodic processes. A single exchange current density is a strong indication that the same active site is operative under HER and HOR conditions since a change in the active center results in a different Butler-Volmer relationship for the kinetics with an altered exchange current density.

Therefore, we arrive at the conclusion that case (1) is met in that the efficient bifunctional hydrogen electrocatalysis, taking platinum as the prototypical example, requires a change in the reaction mechanism. Strictly speaking, for $G_{1}^{\#}$ > $G_{2}^{\#}$ and Δ*G*_TD_ > 0 eV, the Heyrovsky-Volmer mechanism is operative for the HOR with a potential-dependent change of the Tafel slope. Accordingly, the HER has to proceed via the Volmer-Tafel mechanism because otherwise, a potential-dependent switch of the Tafel slope cannot be observed. Similarly, for $G_{1}^{\#}$ < $G_{2}^{\#}$ and Δ*G*_TD_ > 0 eV, the HER is described by the Volmer-Heyrovsky mechanism with a potential-dependent Tafel slope, whereas only the Tafel-Volmer pathway can be observed for the HOR.

Finally, a few caveats of our study and conclusion are discussed. On the one hand, we did not model the Volmer-Tafel mechanism explicitly but rather referred to this pathway in the discussion on a qualitative level to explain the situation in which both the HER and HOR reveal a potential-dependent switching of the Tafel slope. While we can adopt the Volmer-Tafel mechanism for the same data-driven analysis as presented for the Volmer-Heyrovsky mechanism, we want to outline the reasons why there is no need to present a detailed analysis for this pathway herein. If the transition-state free energy of the first Volmer step, $G_{1}^{\#}$, is larger than that of the second Volmer step, $G_{2}^{\#}$, in the Volmer-Tafel mechanism, the HOR reveals potential-dependent switching of the Tafel slope, whereas the HER has a constant Tafel slope of about 118 mV/dec. Similarly, for $G_{1}^{\#}$ < $G_{2}^{\#}$, the opposite scenario is encountered in that the Tafel slope of the HER is potential dependent, whereas a slope of 118 mV/dec. is observed for the HOR. These results do not change if the Tafel step with a transition-state free energy of $G_{3}^{\#}$ is considered in the analysis due to the chemical nature of this step, recalling that a chemical step as the rate-determining step translates to a Tafel slope of infinity.^85^ In summary, even when modeling the Volmer-Tafel mechanism for the reversible HER and HOR by our data-driven approach, the same qualitative picture as illustrated in Figure 4 is observed. Therefore, also the Volmer-Tafel mechanism alone cannot reproduce a potential-dependent switching of the Tafel slope for the HER and HOR both.

Consequently, only the combination of the Volmer-Heyrovsky and Volmer-Tafel mechanisms can give rise to a situation where both the HER and HOR reveal potential-dependent Tafel slopes. Here, the question remains whether this can be illustrated by our data-driven strategy. We would like to emphasize that the FED of the Volmer-Heyrovsky and Volmer-Tafel mechanisms are, besides the transition-state free energy $G_{1}^{\#}$ of the first Volmer step, not interrelated since they consist of different elementary steps. For instance, if the FED of the Volmer-Heyrovsky comprises $G_{1}^{\#}$ < $G_{2}^{\#}$ ($G_{2}^{\#}$ = Heyrovsky step, cf. Figure 4B), the HER reveals potential-dependent switching of the Tafel slope. Consequently, the FED for the Volmer-Tafel mechanism adheres to $G_{1}^{\#}$ > $G_{2}^{\#}$ ($G_{2}^{\#}$ = second Volmer step), and thus, the Tafel-Volmer pathway is met for the HOR with a potential-dependent Tafel slope. This conclusion can directly be made since there is no relationship between the transition-state free energy $G_{2}^{\#}$ (either Heyrovsky or second Volmer step) in the Volmer-Heyrovsky and Volmer-Tafel mechanisms, respectively. In summary, only a change in the reaction mechanism for the HER and HOR can give rise to a potential-dependent Tafel slope for both cathodic and anodic conditions, and therefore we propose this finding as a necessary criterion for efficient bifunctional materials in the hydrogen electrocatalysis.

### Conclusions

In the present manuscript, we discuss the bifunctional hydrogen electrocatalysis, consisting of the HER and HOR. While the application of unitized regenerative proton exchange membrane fuel cell raises the need for efficient bifunctional materials, we discuss their requirements by combining microkinetic modeling with a data-driven analysis to comprehend a general understanding of the reversible hydrogen electrocatalysis on the atomic scale.

Previous experiments by Markovich et al. indicated that platinum, the most active HER and HOR catalyst, reveals potential-dependent Tafel slopes in the HER and HOR both. Studying the bifunctional hydrogen electrocatalysis by our modeling approach, we illustrate that the assumption of a single reaction mechanism for both pathways cannot explain this behavior; rather, the efficient bifunctional hydrogen electrocatalysis requires a change in the reaction mechanism. For instance, if the HER proceeds via the Volmer-Heyrovsky mechanism, the Tafel-Volmer pathway is met for the HOR, and this relation also holds true for the opposite case if the mechanistic descriptions are exchanged.

So far, computational electrochemistry has relied on the adsorption free energy of adsorbed hydrogen, Δ*G*_∗H_, as a descriptor to identify highly active catalysts in the HER and HOR both. While this descriptor has already been challenged recently^31^^,^^34^^,^^86^ we indicate that Δ*G*_∗H_ is not applicable to the identification of bifunctional catalysts for the hydrogen electrode reaction. It is rather required to elucidate the kinetic picture of the reactions, aiming to seek for a switch in the reaction mechanism when moving from anodic to cathodic potential conditions since this is a strong hint for an efficient bifunctional operation.

## STAR★Methods

### Key resources table

**Table:** 

REAGENT or RESOURCE	SOURCE	IDENTIFIER
**Deposited data**		

Our code has been uploaded to Zenodo (https://doi.org/10.5281/zenodo.10368516): https://zenodo.org/records/10368516		

**Software and algorithms**		

Python Details can be found in our code uploaded to Zenodo (https://doi.org/10.5281/zenodo.10368516): https://zenodo.org/records/10368516		

### Resource availability

#### Lead contact

Further information and requests for resources should be directed to and will be fulfilled by the lead contact, Prof. Dr. Kai S. Exner (kai.exner@uni-due.de).

#### Materials availability

This study did not generate new unique reagents.

#### Data and code availability

Data

• Data reported in this paper will be shared by the lead contact upon request. All data is also available in the Python script uploaded to Zenodo (https://doi.org/10.5281/zenodo.10368516) https://zenodo.org/records/10368516.

Code

• A pseudocode is available in this paper’s supplemental information. We have uploaded the full code of our work to Zenodo (https://doi.org/10.5281/zenodo.10368516): https://zenodo.org/records/10368516.
• Any additional information required to reanalyze the data reported in this paper is available from the lead contact upon request.

### Method details

The conclusions rendered in our manuscript rely on the code that is freely accessible on Zenodo (https://doi.org/10.5281/zenodo.10368516) https://zenodo.org/records/10368516.

We have performed microkinetic simulations by steady-state analysis for a two-electron process, such as encountered with the hydrogen evolution and hydrogen oxidation reactions. Equation 19 is the central equation that allows determining current densities, *j*, as a function of applied overpotential, *η*, in dependence of the binding energy of the reaction intermediate, adsorbed hydrogen, and the transition states for the Volmer and Heyrovsky steps. The code for our microkinetic simulations is freely accessible on Zenodo (https://doi.org/10.5281/zenodo.10368516): https://zenodo.org/records/10368516.
