# Supplementary material for: Why efficient bifunctional hydrogen electrocatalysis requires a change in the reaction mechanism
Source: iScience. 2024 Jan 8;27(2):108848. doi: 10.1016/j.isci.2024.108848 (PMC10837630; doi:10.1016/j.isci.2024.108848)
Supplement: Document S1. Figures S1–S4 [file mmc1.pdf]

iScience, Volume *27*

Supplemental information

Why efficient bifunctional hydrogen electrocatalysis requires a change in the reaction mechanism

Samad Razzaq and Kai S. Exner

Supporting Information

Why efficient bifunctional hydrogen electrocatalysis requires a change in the reaction mechanism

Samad Razzaq[1] and Kai S. Exner[1,2,3,*]

[1] *University Duisburg-Essen, Faculty of Chemistry, Theoretical Inorganic Chemistry, Universitätsstraße 5, 45141 Essen, Germany*
[2] *Cluster of Excellence RESOLV, Bochum, Germany*
[3] *Center for Nanointegration (CENIDE) Duisburg-Essen, Duisburg, Germany*

* Corresponding author: kai.exner@uni-due.de

ORCID: 0000-0003-4693-4581 (SR), 0000-0003-2934-6075 (KSE*)

Keywords

hydrogen electrocatalysis; microkinetic modeling; data-driven approach; Tafel slope

S1) Pseudo script:

for U = [0.03,0.20;0.01]:
  for $G_1^{\#}$ = [0.70,0.75,0.75]:
    for $G_2^{\#}$ = [0.70]:
      for ΔG_{TD} = [0,0.40;0.10]:
        for α_1 = [0.50]:
          for α_2 = [0.50]:
            for Γ_{act} = [5×10^14]:
              $G_1^{\#}\ (HER) = G_1^{\#}$
              $G_2^{\#}\ (HER) = G_2^{\#}$
              determine $j_{\mathrm{HER}}(\eta)$ using equation 18
              $G_1^{\#}\ (HOR) = G_2^{\#}$
              $G_2^{\#}\ (HOR) = G_1^{\#}$
              determine $j_{\mathrm{HOR}}(\eta)$ using equation 18
              save all parameters in a DataFrame

Figure S1: Simplified script for our microkinetic modeling study; related to Figures 2-4. Pseudocode of the Python script. The script encodes equation (18) of the main text. While α_1, α_2, and Γ_{act}, $G_2^{\#}$ and Γ_{act} are single-digits numbers, the Python script is written in a manner that any array expansion in the input data set can be implemented readily.

S2) Tafel plots for ΔG_{TD}= 0.20 eV

(a)

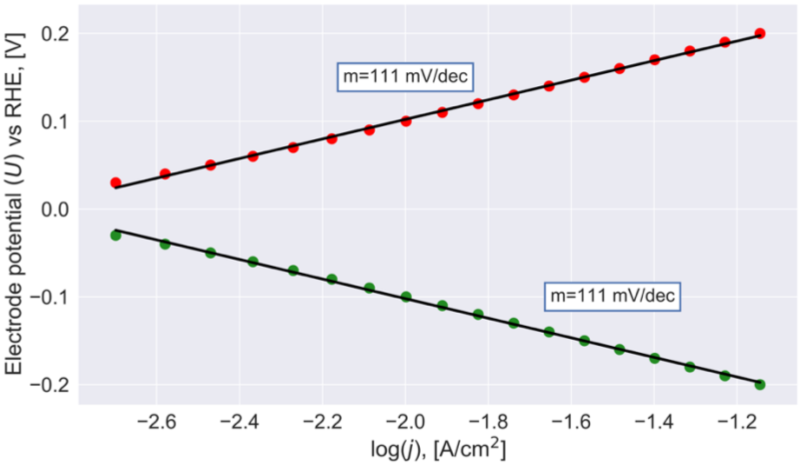

(b)

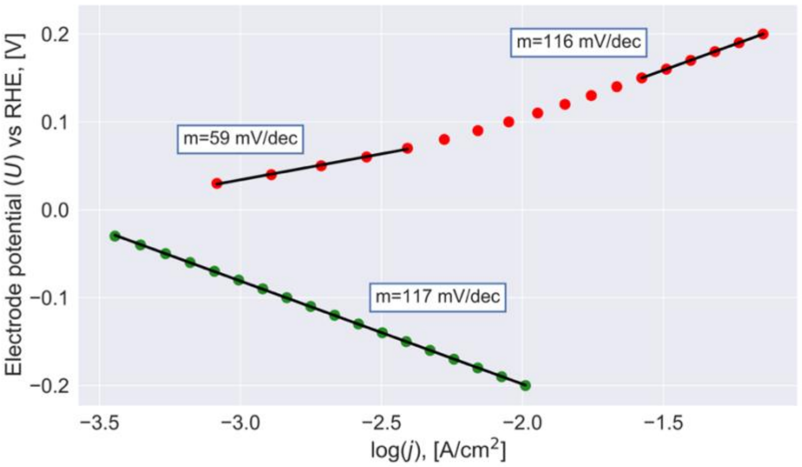

(c)

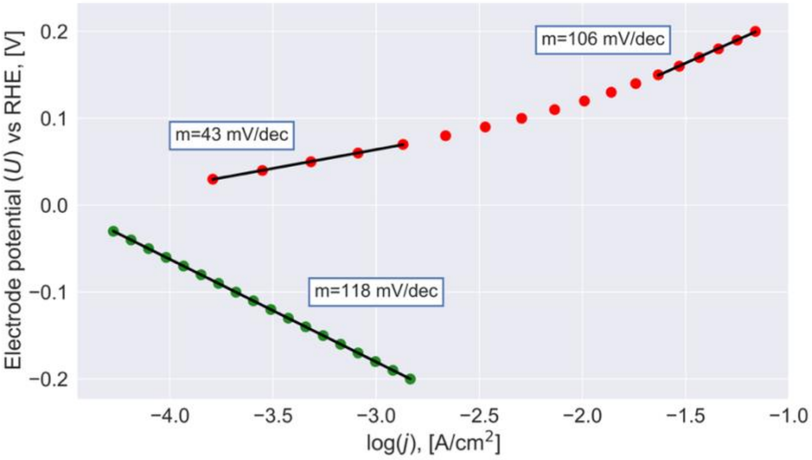

Figure S2: Tafel plots obtained from microkinetic modeling; related to Figure 3. Steady-state analysis (cf. equation (18)) is used to convert the free-energy landscape of **Figure 1** for a data set of various free energies into a Tafel plot. Here, we depict the results for ΔG_{TD}= 0.20 eV and a) $G_1^{\#}$= 0.70 eV, $G_2^{\#}$= 0.70 eV, b) $G_1^{\#}$= 0.75 eV, $G_2^{\#}$= 0.70 eV, c) $G_1^{\#}$= 0.80 eV, $G_2^{\#}$= 0.70 eV. HER and HOR are indicated by green and red data points, respectively. Independent of the actual energetics, a single Tafel slope is observed in the entire potential regime for HER whereas the HOR reveals a change in the Tafel slope for $G_1^{\#} > G_2^{\#}$.

S3) Tafel plots for ΔG_{TD}= 0.30 eV

(a)

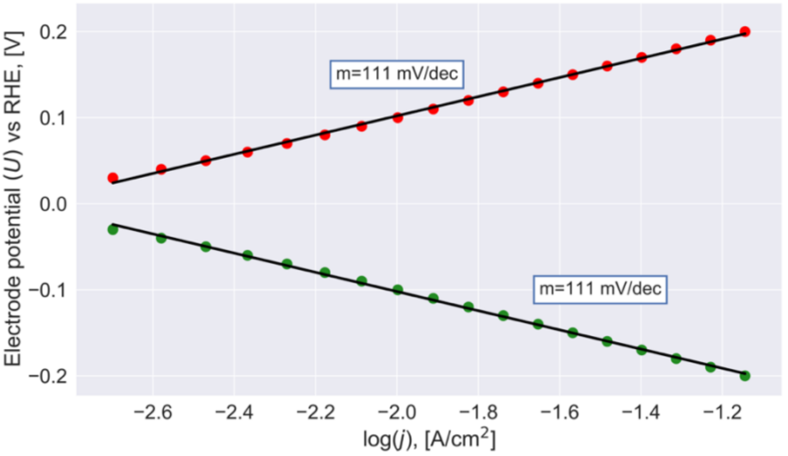

(b)

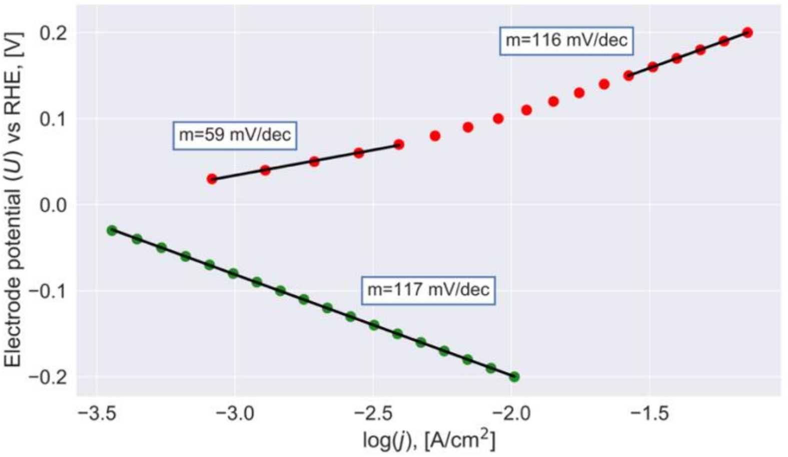

(c)

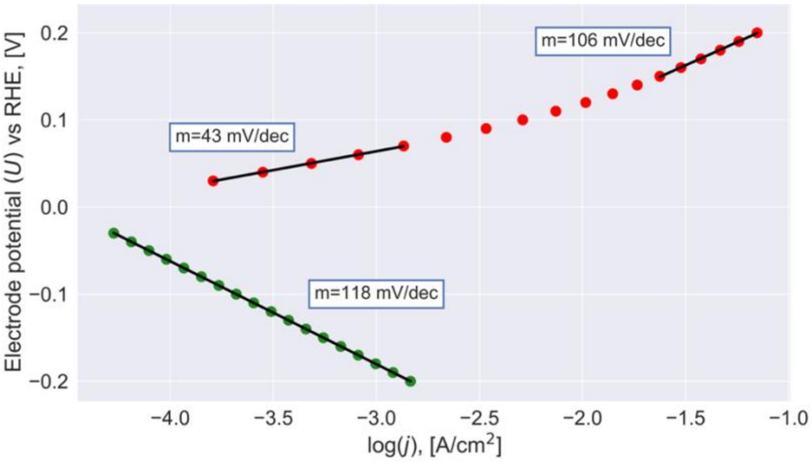

Figure S3: Tafel plots obtained from microkinetic modeling; related to Figure 3. Steady-state analysis (cf. equation (18)) is used to convert the free-energy landscape of **Figure 1** for a data set of various free energies into a Tafel plot. Here, we depict the results for ΔG_{TD}= 0.30 eV and a) $G_1^{\#}$= 0.70 eV, $G_2^{\#}$= 0.70 eV, b) $G_1^{\#}$= 0.75 eV, $G_2^{\#}$= 0.70 eV, c) $G_1^{\#}$= 0.80 eV, $G_2^{\#}$= 0.70 eV. HER and HOR are indicated by green and red data points, respectively. Independent of the actual energetics, a single Tafel slope is observed in the entire potential regime for HER whereas the HOR reveals a change in the Tafel slope for $G_1^{\#} > G_2^{\#}$.

S4) Tafel plots for ΔG_{TD}= 0.40 eV

(a)

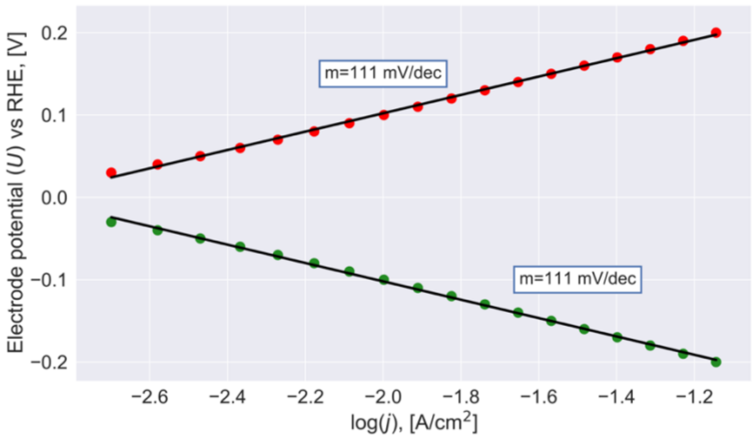

(b)

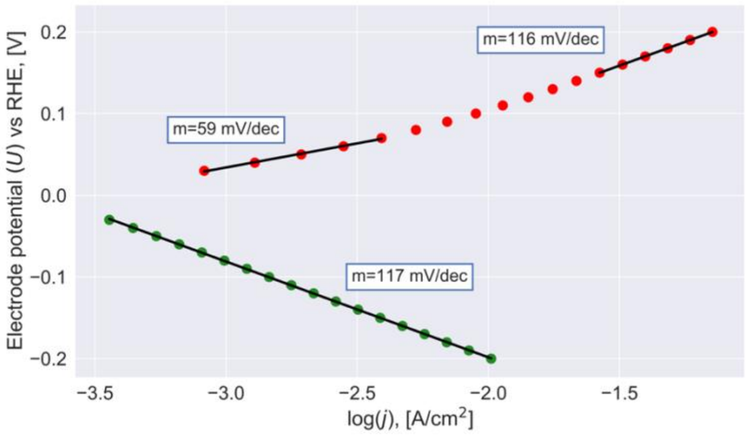

(c)

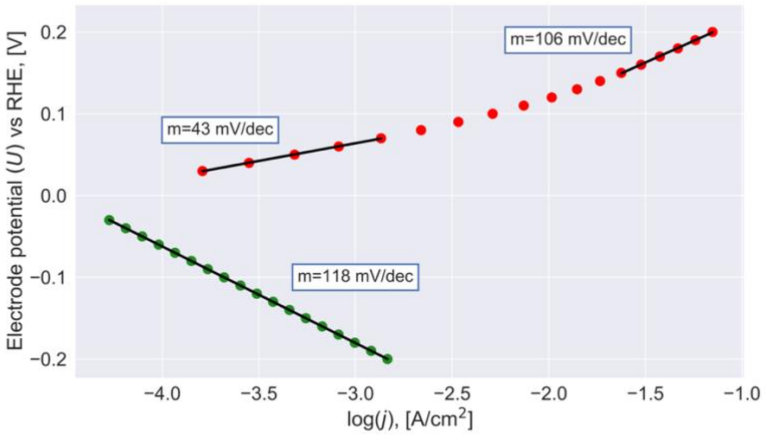

Figure S4: Tafel plots obtained from microkinetic modeling; related to Figure 3. Steady-state analysis (cf. equation (18)) is used to convert the free-energy landscape of **Figure 1** for a data set of various free energies into a Tafel plot. Here, we depict the results for ΔG_{TD}= 0.40 eV and a) $G_1^{\#}$= 0.70 eV, $G_2^{\#}$= 0.70 eV, b) $G_1^{\#}$= 0.75 eV, $G_2^{\#}$= 0.70 eV, c) $G_1^{\#}$= 0.80 eV, $G_2^{\#}$= 0.70 eV. HER and HOR are indicated by green and red data points, respectively. Independent of the actual energetics, a single Tafel slope is observed in the entire potential regime for HER whereas the HOR reveals a change in the Tafel slope for $G_1^{\#} > G_2^{\#}$.
